# Use of extramural ambulatory care curricula in postgraduate medical training

**DOI:** 10.1007/s40037-015-0166-z

**Published:** 2015-04-08

**Authors:** Jaideep S. Talwalkar, D’Juanna Satcher, Teri L. Turner, Stephen D. Sisson, Ada M. Fenick

**Affiliations:** 1 Department of Pediatrics, Yale School of Medicine, 333 Cedar Street, 208086, New Haven, CT 06520-8086 USA; 2 Department of Internal Medicine, Yale School of Medicine, New Haven, CT USA; 3 Department of Pediatrics, Baylor College of Medicine, Houston, TX USA; 4 Center for Research, Innovation, and Scholarship in Medical Education, Baylor College of Medicine, Houston, TX USA; 5 Department of Medicine, Johns Hopkins University School of Medicine, Baltimore, MD USA

**Keywords:** Primary care education, Curriculum development, House officer training, Postgraduate training

## Abstract

**Introduction:**

Extramural curricula developed for the purpose of sharing with other institutions have been designed to improve education on important topics in ambulatory care. We sought to assess the usage rates of these curricula among paediatric, internal medicine, and combined medicine-paediatrics residency programmes in the United States.

**Methods:**

Surveys on aspects of trainee continuity clinic were sent to paediatric and medicine-paediatrics programme directors in 2012. Surveys contained an item asking respondents about their use of extramural ambulatory care curricula. Since no similar recent data were available for internal medicine, and to verify the accuracy of the paediatric survey data, we queried the editors of four widely used curricula for subscription information. Descriptive and inferential statistics were calculated.

**Results:**

Responses from paediatric programmes indicated that 48 of 111 (43 %) were using an extramural curriculum, compared with 39 of 60 (65 %) medicine-paediatrics programmes (*p* = 0.007). Editor query revealed a collective subscription rate of internal medicine programmes (300 of 402, 75 %), which was greater than the subscription rate of paediatric programmes (90 of 201, 45 %) (*p* < 0.001).

**Discussion:**

Training programmes in paediatrics, internal medicine, and combined medicine-paediatrics utilize extramural curricula to guide education in ambulatory care, but internal medicine and medicine-paediatrics programmes employ these curricula at greater rates than paediatric programmes.

## Background

Paediatrics and internal medicine residency training programmes in the United States provide exposure to content areas in both the outpatient and inpatient settings as outlined by speciality certification boards and the Accreditation Council for Graduate Medical Education (ACGME) [1]. Residents spend most of their time in the hospital and report feeling inadequately prepared to provide outpatient care [2, 3]. Additionally, studies have confirmed deficiencies in knowledge among residents related to common topics in ambulatory care for both children and adults [2, 4]. Thus, there is a clear need for effective educational resources for use in the outpatient setting [2].

Internal medicine and paediatrics are two fields that remain, in practice, strongly ambulatory. Historically, residency programmes have struggled to provide training in ambulatory care; training remains inpatient-centric [2, 3]. Programmes have continued to find ways to shift the emphasis to improve training in ambulatory care, and the development of ambulatory care curricula is part of the response to address the imbalance [2, 5].

The development of educational resources is time consuming, labour-intensive, and expensive [6]. The sharing of curricular resources is becoming an increasingly common phenomenon among medical educators through use of ‘extramural curricula,’ those which have been developed by other institutions or have been developed at one’s home institution for the purpose of sharing [6−8]. The use of extramural curricula permits more efficient use of faculty time [6], shared expense towards curriculum development, and evaluation via multi-centre trials [7].

Extramural curricula have been designed to improve education on important topics in ambulatory care for children [5] and adults [7]. Training programmes use them when no internal ambulatory care curriculum exists, to replace an existing curriculum, or to augment current local offerings [9]. These curricula have resulted in improvements in resident participation in learning, satisfaction, confidence in clinical skills [5], and knowledge [7]. They are widely used among internal medicine training programmes to enhance exposure to ambulatory care topics [2, 10].

In the United States, residencies in paediatrics and internal medicine share similarities in structure (e.g., duration of training, ambulatory care requirements) and goals (e.g., health promotion, disease prevention, exposure to common conditions seen in practice) [1] so a similar solution to the challenge of educating trainees in the outpatient setting would seem reasonable. However, implementation rates of extramural ambulatory care curricula among paediatric training programmes in the United States are unknown.

The purpose of this study was to compare usage rates of extramural ambulatory care curricula among paediatric residency programmes in the United States with usage rates among internal medicine programmes, including combined internal medicine-paediatrics programmes. We hypothesized that differences in usage rates would exist among programme types.

## Methods

Two independent surveys designed to characterize various aspects of the ambulatory experience were sent to paediatric and medicine-paediatrics programme directors, respectively, in the spring of 2012. As part of a project at Baylor College of Medicine on paediatric residency curricula, surveys were sent electronically via SurveyMonkey to 198 of 199 ACGME accredited paediatric residency programmes, excluding Baylor itself. The survey was sent to a programme’s clinic director if that person could be identified; otherwise it was sent to the programme director. The survey consisted of 13 items regarding the residency programme’s demographics and characteristics of their curriculum, one of which related to the use of extramural ambulatory care curricula. This item stated, ‘If you have purchased (a formal standardized curriculum for continuity clinic) please specify which one.’ Four choices were provided: ‘Johns Hopkins; University of Chicago; Yale; Other (specify).’

The Medicine-Pediatrics Program Directors Association (MPPDA) distributes an annual survey to each medicine-paediatrics programme listed in the National Residency Match Program database. The survey is designed to understand programmatic structure, support, curriculum, and communication with regulatory agencies. The 2012 survey was distributed via SurveyMonkey to 79 medicine-paediatrics programme directors with email addresses on file with the MPPDA, a list that is nearly identical to medicine-paediatrics residency programmes in the Electronic Residency Application Service (ERAS) database. The survey consisted of 88 items, one of which related to the use of extramural ambulatory care curricula. This item stated ‘Does your medicine-paediatrics programme use any of the ambulatory curricula developed by: Johns Hopkins; Vanderbilt; Yale; Other (specify); I have not heard of any ambulatory curricula.’

The most recent published data on usage rates of extramural ambulatory care curricula for categorical internal medicine training programmes date back to 2005 [10] and 2006 [2]. These dated figures were deemed unsuitable for comparison. In order to determine current usage rates, we queried the editors of the Yale Office-Based Medicine Curriculum and the Johns Hopkins Internet Learning Center Internal Medicine Curriculum, the two extramural curricula commonly known to be widely used among internal medicine residency programmes [10]. To verify the accuracy of this data collection method for comparison purposes to the survey data from paediatric and medicine-paediatrics programmes, we also collected subscription rate information for the Yale Primary Care Pediatrics Curriculum and Johns Hopkins Internet Learning Center Pediatrics Ambulatory Care Curriculum through direct query.

Data were analyzed with SAS 9.3 (Cary, NC). A two-sample test for proportions was used to compare curricular utilization rates between the groups. Significance was defined as *p* ≤ 0.05.

## Results

Surveys were returned by 111 of 198 (56 %) paediatric and 63 of 79 (80 %) medicine-paediatrics residency programmes. Of the medicine-paediatrics survey respondents, 60 answered the item related to extramural ambulatory care curricula. Responses from paediatric programmes indicated that 48 of 111 (43 %) were using an extramural curriculum, compared with 39 of 60 (65 %) medicine-paediatrics programmes (*p* = 0.007). Most paediatric programmes utilizing an extramural curriculum indicated using the Yale (23/48, 48 %) or Johns Hopkins (22/48, 46 %) curricula. One programme indicated utilizing both curricula.

There are 402 categorical internal medicine residency programmes listed in the ERAS database. In personal correspondence, the editors of the Yale Office-Based Medicine Curriculum and Johns Hopkins Internet Learning Center Internal Medicine Curriculum indicated that 130 and 170 internal medicine residency programmes had active subscriptions in 2012.

The query to the editors of the Yale Primary Care Pediatrics Curriculum and Johns Hopkins Internet Learning Center Pediatrics Ambulatory Care Curriculum revealed 42 and 48 paediatric programme subscriptions, respectively, in 2012, collectively representing 45 % of the 201 categorical paediatric residency programmes listed in the ERAS database. Numbers of medicine-paediatrics programme subscriptions were unavailable from all curricular editors.

The collective subscription rate of internal medicine programmes (300 of 402, 75 %) was greater than the subscription rate of paediatric programmes (90 of 201, 45 %) (*p* < 0.001) (Table 1).

**Table 1:** Extramural ambulatory care curriculum utilization

	Paediatrics	Med-Peds	Medicine	*p* ^a^
Survey respondent indicating use of extramural ambulatory care curriculum	48/111 (43 %)^b^	39/60 (65 %)	NA	0.007
Subscription query of curriculum editors^c^	90/201 (45 %)^d^	NA	300/402 (75 %)^e^	< 0.001

*Med-Peds* Medicine-Paediatrics

^a^Two-sample test for proportions

^b^Identified by respondents as 23 Yale, 22 Johns Hopkins, and 4 other; one respondent indicated using both Yale and Johns Hopkins paediatric curricula

^c^Numerator is positive responses from individual curriculum editors of the Yale Office-Based Medicine Curriculum, Yale Primary Care Pediatrics Curriculum, and Johns Hopkins Internet Learning Center; denominator the number of programmes listed in the Electronic Residency Application Service database as of March 2013

^d^Yale editors identified 42 programmes, Johns Hopkins 48

^e^Yale editors identified 130 programmes, Johns Hopkins 170

## Discussion

Training programmes in paediatrics, internal medicine, and combined internal medicine-paediatrics utilize extramural curricula to guide education in ambulatory care, but internal medicine and medicine-paediatrics programmes employ these curricula at greater rates than do paediatric programmes. The difference in utilization rates of extramural ambulatory care curricula between these training environments has not been previously identified.

While specific content areas differ between paediatrics and internal medicine, the structures of the training environments in the United States are similar. Residents split time between inpatient and outpatient settings, and must spend at least 108 (paediatrics) and 130 (internal medicine) half-day sessions engaged in a longitudinal outpatient experience spaced out over three years. This experience must provide residents with exposure to common conditions seen in ambulatory care settings, under the direct supervision of faculty [1]. It is unknown why these parallel training environments have embraced a common solution with such disparate usage rates, though many possible explanations exist.

Extramural ambulatory care curricula for internal medicine have been available for over 20 years [9], while similar curricula geared towards paediatric training are a newer phenomenon [5]. Sisson and colleagues describe growth in usership of the Johns Hopkins Internet Learning Center Internal Medicine Curriculum from 24 programmes in 2001 [7], the first year the curriculum was made available outside of their centre, to 67 programmes in 2006 [9], to 170 programmes in 2012. It is unknown whether the growth in usership over time is due to increased awareness as trainees take their knowledge of teaching resources with them to new programmes after graduation, accumulation of data indicating educational effectiveness, or other factors. The bulk of the current literature pertains to the internal medicine curricula [2, 7, 9, 10], so if strength of data is a major driver for prospective users, more outcomes data are needed in support of paediatric extramural curricula.

The internal medicine curricula cover more topics, which may make them more appealing to prospective users. The most recent versions of the Yale and Johns Hopkins internal medicine curricula offer 144 and 41 modules, respectively, compared with 86 and 25 for their paediatric counterparts. It is unlikely that paediatric educators in the United States are fundamentally more reluctant to utilize extramural curricula, given that uptake for shared educational solutions in the inpatient paediatric environment has been well described [11].

The fact that internal medicine and medicine-paediatrics programmes use extramural curricula at higher rates than paediatrics programmes may be indicative of the application of a curricular solution across graduate medical education (GME) disciplines. Combined training programmes may be in a unique position to bring educational solutions to their categorical counterparts. Medical educators should be aware of effective curricular solutions across geographies and GME programmes as enhanced digital availability improves access [6, 8].

## Limitations and strengths

We utilized two different methods to determine the extramural curriculum usership rates for the three types of residency programmes, though we limited our calculations to numbers obtained through similar methodologies only. We believe that the results obtained reflect actual usage rates since the paediatric usage rate obtained via survey of clinic directors was nearly identical to that obtained through direct inquiry of curriculum editors. Additionally, survey response rates differed for paediatrics and medicine-paediatrics programmes. The higher response rate to the medicine-paediatrics survey may have been attributable to its being part of a familiar annual exercise among members of a professional organization, in which all members have a vested interest.

Our exploratory study is the first to identify differences in utilization rates of extramural ambulatory care curricula among parallel GME programmes. Our future research will examine the factors that impact subscription decisions among programme types, including exploration of the theories posed here.

## Conclusion

The usage rate of extramural ambulatory care curricula among paediatric residency programmes was significantly lower than usage rates in medicine-paediatrics and internal medicine programmes. Given the similarities in training structure, paediatric educators may wish to look to curricular solutions utilized by internal medicine programmes. Combined training programmes may be uniquely situated to offer educational solutions across training disciplines.

### Funding

None.

## References

[1] Accreditation Council for Graduate Medical Education. ACGME Program Requirements for Medical Specialties. http://www.acgme.org/acgmeweb/tabid/368/ProgramandInstitutionalAccreditation/MedicalSpecialties.aspx. Accessed 27 June 2014.

[2] Sisson SD, Dalal D (2011). Internal medicine residency training on topics in ambulatory care: a status report. Am J Med.

[3] Freed GL, Dunham KM, Switalski KE, Jones MD, McGuinness GA (2009). Research Advisory Committee of the American Board of Pediatrics. Recently trained general pediatricians: perspectives on residency training and scope of practice. Pediatrics.

[4] Robers KB, Starr S, DeWitt TG (2002). Resident preparedness for practice: a longitudinal cohort study. Ambul Pediatr.

[5] Talwalkar JS, Fenick AM (2011). Evaluation of a case-based primary care pediatric conference curriculum. J Grad Med Educ.

[6] Candler CS, Uitjdehaage SHJ, Dennis SE (2003). Introducing HEAL: the Health Education Assets Library. Acad Med.

[7] Sisson SD, Hughes MT, Levine D, Brancati FL (2004). Effect of an internet-based curriculum on postgraduate education: a multicenter trial. J Gen Intern Med.

[8] Reynolds RJ, Candler CS (2008). MedEdPORTAL: educational scholarship for teaching. J Cont Educ Health Prof.

[9] Sisson SD, Rastegar DA, Rice TN, Hughes MT (2007). Multicenter implementation of a shared graduate medical education resource. Arch Intern Med.

[10] Key NA, Al-Hihi E, Whittle J (2005). To buy or build: deciding on an ambulatory resident’s curriculum. SGIM Forum.

[11] Starmer AJ, O’Toole JK, Rosenbluth G (2014). Development, implementation, and dissemination of the I-PASS handoff curriculum: a multisite educational intervention to improve patient handoffs. Acad Med.

